# A Case of Cholecystitis Camouflaging Cholangiocarcinoma

**DOI:** 10.7759/cureus.55448

**Published:** 2024-03-03

**Authors:** Sreshta Paranji, Rathnamitreyee Vegunta, Christine Pellegrino

**Affiliations:** 1 Internal Medicine, Westchester Medical Center, Valhalla, USA; 2 Hematology/Oncology, Westchester Medical Center, Valhalla, USA

**Keywords:** cholangiocarcinoma, palliative care, duodenal obstruction, elevated ca 19-9, adenocarcinoma, acute calculus cholecystitis

## Abstract

Cholangiocarcinoma is a malignancy that is hard to detect and resect, due mostly to its location as well as a lack of current screening tests. When found, it is often in the advanced stage as patients are usually asymptomatic during the early course of the disease; the overall prognosis is modest in patients diagnosed at this stage. Here, we discuss the case of a 48-year-old female with no significant past medical history or family history who presented to our hospital with symptoms of acute cholecystitis with a supporting ultrasound. She proceeded to get a laparoscopic cholecystectomy for the same, but an ensuing workup and pathology revealed advanced-stage cholangiocarcinoma. The patient ultimately opted for palliative care given her poor prognosis.

## Introduction

Cholangiocarcinomas account for fewer than 5% of all gastrointestinal malignancies, but biliary tract cancer incidence and mortality globally have increased over the last 20 years [1]. The incidence was found to be 0.35-2.00 per 100,000 annually in the Western world, and it has shown a consistent and steady rise in countries like the United Kingdom and the United States at 0.1-0.6 per 100,000 over the last 30 years. However, other countries have shown great clustering of cholangiocarcinoma cases like Japan and northern India [2]. In the Western hemisphere, one of the most common risk factors for the development of cholangiocarcinoma is the inflammatory condition of primary sclerosing cholangitis, with or without comorbid conditions [3]. In the Eastern part of the world, parasitic liver diseases are proven to have an association.

Factors that contribute to shorter overall survival for cholangiocarcinoma, making it imperative to diagnose and/or detect early, include larger tumor size, presence of multiple tumors, lymph node metastasis, and vascular invasion [4]. Presenting symptoms of cholangiocarcinoma often include jaundice, abdominal pain, generalized itching, weight loss, and/or fever [5]. This largely depends on the location of the tumor, as intrahepatic versus extrahepatic cholangiocarcinomas manifest differently. Intrahepatic cholangiocarcinomas are less likely to cause jaundice when compared with extrahepatic cholangiocarcinomas. Occasionally, these symptoms can be accompanied by abnormalities in liver function tests and/or liver enzyme tests which can be indicative of cholangiocarcinoma. It can often portray an obstructive jaundice picture with normal transaminase levels but elevated alkaline phosphatase and direct bilirubin. Diagnosing and managing cholangiocarcinoma is complex. This case of cholangiocarcinoma found in an advanced stage illustrates the challenges associated with diagnosing this at an early enough stage for prolonged survival with treatment.

## Case presentation

A 48-year-old woman with no significant past medical history presented to our hospital with a two-day history of right upper quadrant pain with associated nausea and vomiting. The patient denied any cigarette smoking history or alcohol use. A review of systems on admission was negative for fevers, chills, weight loss, weakness, or fatigue. Physical examination was significant only for right upper quadrant abdominal tenderness. Labs were significant only for a mildly elevated white blood cell count (Table 1). An ultrasound of the abdomen showed cholelithiasis, gallbladder wall thickening, and a common bile duct measuring 5 mm in diameter.

**Table 1:** Laboratory results on initial presentation.

Lab parameter	Patient’s valve	Reference range
White blood cell count	15 k/mm^3^	4.8–10.8 k/mm^3^
Aspartate transaminase	32 U/L	4–35 U/L
Alanine transaminase	47 U/L	6–55 U/L
Alkaline phosphatase	139 U/L	40–150 U/L
Total bilirubin	0.6 mg/dL	0.2–1.3 mg/dL

**Figure 1:**
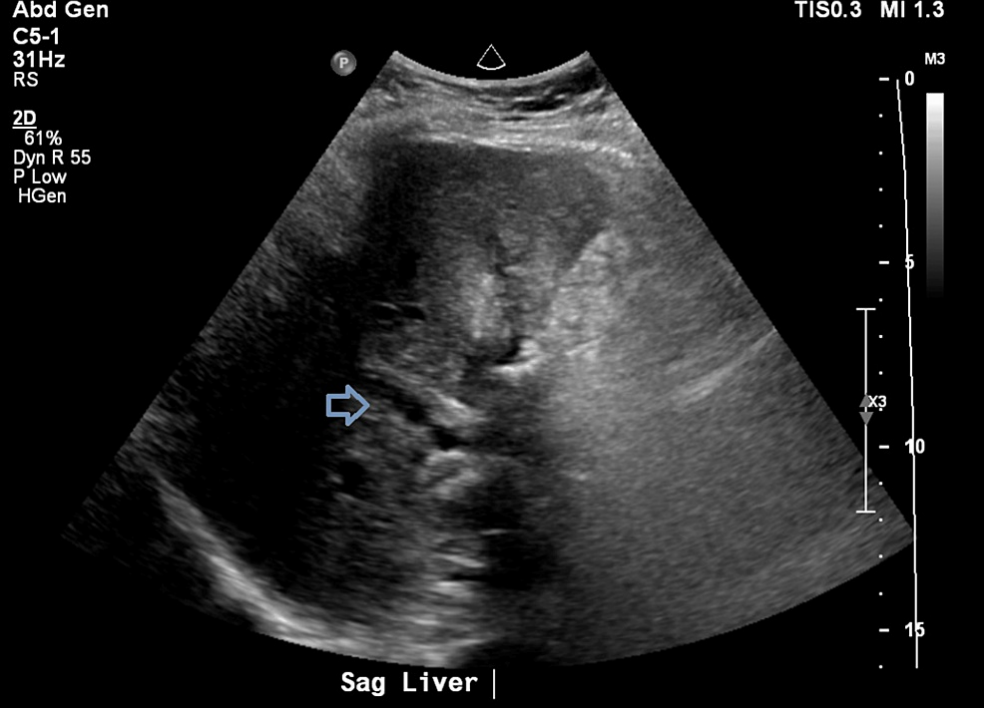
Ultrasound imaging on return admission showing biliary ductal dilation.

**Figure 2:**
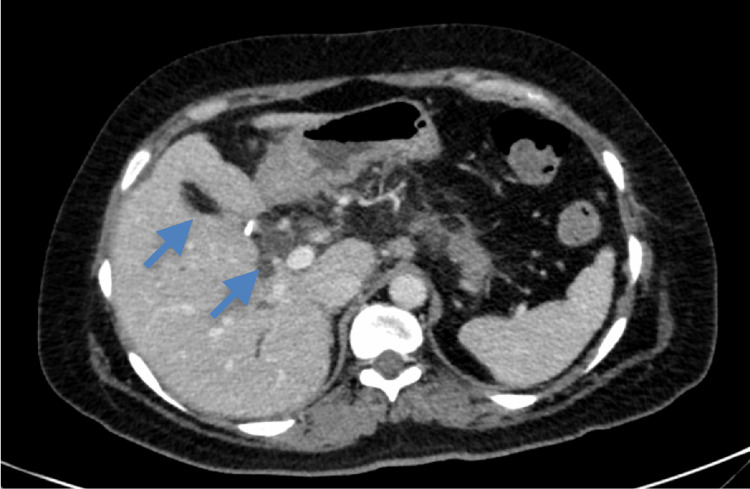
CT scan of the abdomen/pelvis showing biliary duct abnormalities.

**Figure 3:**
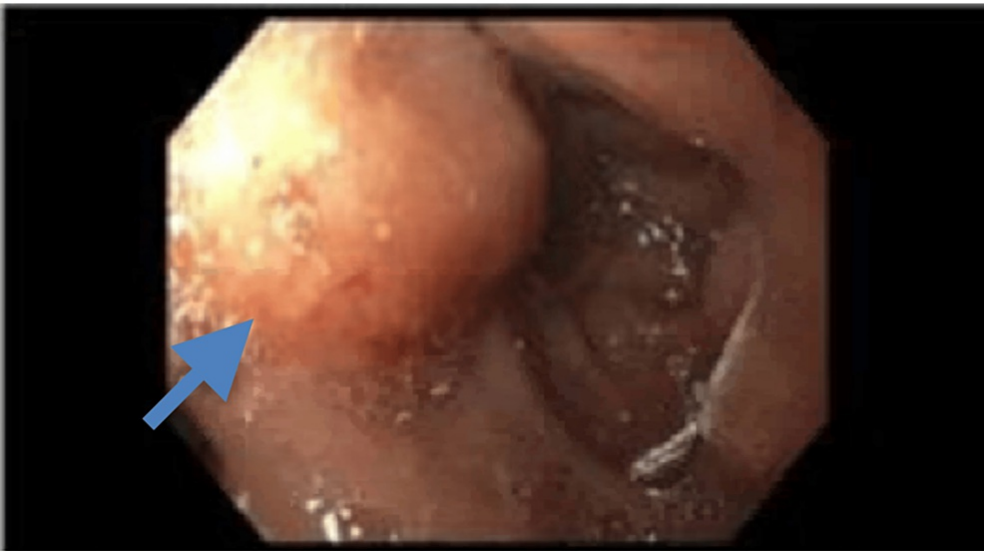
Duodenal bulb showing extrinsic compression, unable to advance probe to complete endoscopic retrograde cholangiopancreatography.

Thus, the patient underwent laparoscopic cholecystectomy as the concern was cholecystitis. Postoperatively, the patient recovered without complication and was discharged within three days with a plan for close follow-up. However, the following surgical pathology resulted in poorly differentiated adenocarcinoma with invasion into muscle. As the patient was at least T1b stage given the pathology read, the patient was planned for hepatic resection and lymphadenectomy. Meanwhile, the patient returned to the hospital three weeks later with worsening right upper quadrant pain, new jaundice, and pruritus. She was afebrile with physical examination findings significant for jaundiced skin and diffuse abdominal tenderness. Labs were significant for elevated transaminases as well as a newly elevated cancer antigen (CA) 19-9 value of 5,538.2 U/mL (reference range <35 U/mL). This time, the ultrasound of her abdomen revealed intrahepatic and extrahepatic biliary ductal dilation with heterogeneous echotexture of the liver (Figure 1). A CT scan of the abdomen/pelvis was significant for focal luminal narrowing of the mid to distal common bile duct (Figure 2). This prompted a magnetic resonance cholangiopancreatography, which revealed thickening and enhancement of the common bile duct. The patient subsequently underwent an endoscopic ultrasound (EUS) that revealed extrinsic compression in the duodenal bulb causing narrowing of the lumen with poor distensibility (Figure 3), so an endoscopic retrograde cholangiopancreatography was unable to be performed.

However, shortly after, she developed duodenal obstruction with severe vomiting and was unable to tolerate oral (PO) intake. Due to this and her young, otherwise healthy status, the patient was taken to the operating room with the intention of a Whipple procedure or a pancreaticoduodenectomy. This generally involves removing the head of the pancreas, the first part of the small intestine, the gallbladder, and the bile duct. In this patient’s case, it would involve removing the head of the pancreas and the first part of the small intestine given the recent cholecystectomy. Unfortunately, in the operating room, the surgeons found that she had diffuse carcinomatosis involving the entire porta hepatis, the anterior surface of the stomach, as well as the gastrohepatic ligament. She had enlarged, involved lymph nodes all along the hepatic artery and a peritoneal deposit on the retroperitoneal side of the duodenum. A frozen section was concerning for adenocarcinoma immediately in the operating room, so the decision was made to not attempt resection but rather to perform a gastrojejunal anastomosis for tolerating PO intake. A duodenal bulb biopsy was obtained and the resulting pathology was significant for tumor cells with predominantly solid architecture and rare gland formation present in the lamina propria of duodenal mucosa; markers were positive for CK7 and CK19 and negative for CDX2, consistent with pancreaticobiliary origin. Regardless, given the diffuse metastasis and poor prognosis, the patient opted for palliative care.

## Discussion

The treatment and prognosis of cholangiocarcinoma depend on the resectability of the tumor and the staging. If the tumor is resectable with negative tissue margins, the resection offers the only chance of a potential cure. Among patients who undergo potentially curative resection for cholangiocarcinoma, long-term outcomes vary according to the location and stage of the primary lesion, extent of surgery, associated comorbidities, and treatment-related complications. The main prognostic factors are histologic margin status and lymph node involvement. In a retrospective review of 137 cases of resected extrahepatic cholangiocarcinoma, five-year survival rates for those with node-negative versus node-positive disease were 38% versus less than 10%, respectively [6]. The number of involved lymph nodes also influences outcomes. In a study of 320 patients undergoing resection of a perihilar cholangiocarcinoma, survival for patients with multiple nodal metastases was significantly worse than for those with a single metastasis (12% versus 28% at five years) [7]. Five-year survival rates are also substantially better with clear as opposed to histologically involved margins (19-47% versus 0-12%, respectively). In keeping with this, the most encouraging results, particularly with perihilar cholangiocarcinomas, come from reports that utilize expanded resection criteria and more extensive surgical procedures which increase the likelihood of negative resection margins [8,9].

However, the majority of cholangiocarcinomas, when diagnosed, present as an unresectable disease, and the five-year survival is found to be 10-25% in advanced cases [5]. Guidelines from the National Comprehensive Cancer Network (NCCN) state that in patients with locally advanced, unresectable, or metastatic cancer, the standard treatment remains gemcitabine/cisplatin combination chemotherapy irrespective of intrahepatic or extrahepatic location [10]. Recently, for advanced cholangiocarcinoma, guidelines have changed regarding systemic therapy. The phase III TOPAZ-I study of the addition of the programmed death-ligand 1 inhibitor durvalumab to gemcitabine and cisplatin showed improved overall survival in the first-line treatment of patients in this population [11], as the estimated 24-month overall survival rate was 24.9% in the patients who received durvalumab with standard gemcitabine and cisplatin combination chemotherapy compared to 10.4% in patients who received placebo with the same combination chemotherapy regimen. Another phase III trial, KEYNOTE-966, showed benefit in the addition of pembrolizumab to gemcitabine plus cisplatin in patients with unresectable, locally advanced, or metastatic biliary tract cancer with an improved overall survival of 12.7 versus 10.9 months [12]. For patients with advanced or metastatic HER2-positive cholangiocarcinoma who progress on the aforementioned therapies, trastuzumab plus tucatinib can be employed as another line of therapy, according to a recent study from November 2023 [13] showing median progression-free survival of six months.

The above-mentioned patient presented for the first time with symptoms classically associated with cholecystitis. She noted that before this she was feeling well. She had no personal medical history, nor a family history of cancer. An entire review of systems completed with her at the time was positive for only abdominal pain, nausea, and vomiting. The ensuing surgery to remove her gallbladder due to the cholecystitis showed us the underlying pathology and disease. Interestingly, at this point, the patient was already advanced with metastasis despite no prior symptoms and only acute symptoms of cholecystitis. When the resulting pathology showed adenocarcinoma with invasion into the muscle, the patient was planned for another surgical procedure. It was only when she returned within one month of discharge feeling worse that she returned with symptoms that are more classically associated with biliary tract cancer. Finally, given the aforementioned pathological tumor markers, the CA 19-9 value, and the EUS showing extrinsic compression at the duodenum, she was appropriately diagnosed with cancer likely to be pancreaticobiliary in origin. Her widespread metastasis found in the operating room and deteriorating functional status led her to be a poor candidate for chemotherapy, as the patient herself felt she would not be able to tolerate it. Had her cholangiocarcinoma been localized and resectable, the patient could have been a candidate for further therapies, including, but not limited to, radiation therapy and/or concurrent chemotherapy.

## Conclusions

Cholangiocarcinoma often presents with symptoms related to obstruction of the biliary tract. Cholecystitis is a rare presentation of the disease and shows that the presentation is quite heterogeneous. Given the rarity and unfavorable outcomes associated with this disease, early diagnosis and a high index of suspicion are crucial. Furthermore, if an incidental finding of adenocarcinoma is found on pathology, a timely workup in the form of imaging should immediately follow. Ultimately, as long-term survival depends on the effectiveness of surgical therapy, attempts to diagnose tumors at an early stage might influence the outcome. The association between gallstone disease and cholangiocarcinoma may reveal a method to improve prognosis by providing an opportunity for earlier diagnosis and treatment. Ultimately, we can use this case as an example to remain prudent in differentials for patients presenting with symptoms of cholecystitis.
